# RETRACTION: CNB‐001 a Novel Curcumin Derivative, Guards Dopamine Neurons in MPTP Model of Parkinson’s Disease

**DOI:** 10.1155/bmri/9864670

**Published:** 2026-05-22

**Authors:** BioMed Research International

RETRACTION: R. L. Jayaraj, N. Elangovan, K. Manigandan, S. Singh, S. Shukla, “CNB‐001 a Novel Curcumin Derivative, Guards Dopamine Neurons in MPTP Model of Parkinson’s Disease,” *BioMed Research International*, 2014, 236182, https://doi.org/10.1155/2014/236182.

The above article, published online on 17 June 2014 in Wiley Online Library (wileyonlinelibrary.com), has been retracted by agreement between the journal Editor‐in‐Chief, Yoel Klug; and John Wiley & Sons Ltd. The retraction has been agreed due to concerns identified with Figures 4, 5 and 6. The concerns with Figures 5 and 6 were identified on PubPeer [1], and subsequent investigation by the Publisher identified the duplications in Figure 4. The following is a summary of the concerns:

• In Figure 4a, there are multiple instances of unexpected, repeated regions within panel D
• In Figure 4a, there is an unexpected, repeated region between panels A and D
• In Figure 4a, there is an unexpected, repeated region within panel A
• In Figure 5a, there are multiple instances of unexpected, repeated regions within panels E, G and H
• In Figure 6a, the DAT bands appear highly similar to the GDNF bands shown in Figure 6a of a previous publication by different authors [2]
• In Figure 6c, the DAT bands appear highly similar to the VMAT‐2 bands shown in Figure 7a of a previous publication by different authors [2]
• In Figure 6c, the TH bands appear highly similar to the TH bands shown in Figure 7a of a previous publication by different authors [2]
• In Figure 6c, VMAT‐2 bands appear highly similar to the TH bands shown in Figure 7a of a publication by different authors [3]
• In Figure 6c, the ß‐actin bands appear identical to the ß‐actin bands shown in Figure 7 of [4]
• In Figure 6a, the ß‐actin bands appear highly similar to the the ß‐actin bands shown in Figure 6a of [5]

After assessment of the author’s response and the concerns by the editorial board, the results can no longer be considered reliable, and the article is therefore retracted.

The authors did not respond to the retraction.
